# The Next Session

**Published:** 1857-04

**Authors:** 


### EDITORIAL AND MISCELLANEOUS.

### THE NEXT SESSION.

As will be seen from the announcement, the next course of lectures in the Atlanta Medical College will commence on the first Monday in May, and from the information we are receiving, it is manifest that there will be a large class.

The facilities for teaching have been greatly increased since the last course, especially in the Anatomical department, a large supply of fine material having been secured, by which we mean to demonstrate that Anatomy can be as well taught here, as in any winter school.

There has been a continuance of the friendly spirit manifested upon the part of the Medical Colleges generally; in the outset of this enterprise, and at the same time, a continuance of hostile demonstrations from those who were an exception to the general course upon this subject, and we are sorry to say that the opposition to the success of this Institution has not been of that open and manly character which can at least be respected, if not approved.

In conclusion, we would remind those who are engaged in *secret* movements against this College, that there is the possibility of such a thing, as the sword which they have drawn, entering into their own hearts.

